# The working disadvantaged: the role of age, job tenure and disability in precarious work

**DOI:** 10.1186/s12889-020-09938-1

**Published:** 2020-12-10

**Authors:** Arif Jetha, Kathleen A. Martin Ginis, Selahadin Ibrahim, Monique A. M. Gignac

**Affiliations:** 1 Institute for Work & Health, Suite 1800, 480 University Avenue, Toronto, ON M5A 1S5 Canada; 2 Dalla Lana School of Public Health, University of Toronto, Toronto, ON Canada; 3 Department of Medicine, Division of Physical Medicine & Rehabilitation, University of British Columbia, Vancouver, BC Canada; 4 School of Health and Exercise Sciences, University of British Columbia, Kelowna, BC Canada; 5 Centre for Chronic Disease Prevention and Management, Southern Medical Program, University of British Columbia, Kelowna, Canada; 6 Krembil Research Institute, Toronto, ON Canada

**Keywords:** Precarious working conditions, Disability, Job tenure, age, multigroup modeling

## Abstract

**Background:**

Precarious work is an increasingly common characteristic of industrialized labor markets that can widen health inequities, especially among disadvantaged workforce segments. Study objectives are to compare precarious employment in workers with and without disabilities, and to examine the modifying effect of disability in the relationships between age, job tenure and precarious work.

**Methods:**

Employed Canadians with (*n* = 901) and without disabilities (*n* = 901) were surveyed on exposure to precarious working conditions. Information on age and job tenure were collected from respondents along with sociodemographic, health and work context details. Multivariable logistic models examined the association between disability and precarious work. Also, multigroup probit models examined precarious work for young (18-35 yrs), middle-aged (36-50 yrs) and older adults (> 50 yrs) and job tenure and was stratified by participants with and without disabilities.

**Results:**

Almost equal proportions of young, middle-aged and older participants were recruited. Mean job tenure of participants was 9.5 years (SD = 9.0). Close to one-third of participants reported working precariously. At the multivariable level, a disability was not associated with working precariously. However, multigroup modelling indicated that disability was a significant effect-modifier. Older adults with a disability had a 1.88 times greater odds of reporting precarious work when compared to young adults (OR = 1.88, 95%CI 1.19, 2.98). When reporting a disability, longer job tenure was related to a 0.95 times lower odds of precarious work (OR = 0.95 95%CI 0.93, 0.98). The relationship between age and job tenure was not significant for those not reporting a disability.

**Discussion:**

Precarious work has the potential to affect workers with and without disabilities. For those with a disability, being an older adult and/or a new worker can contribute to a greater likelihood of being employed precariously. Policies and programs can be recommended to address precarious working conditions and related health inequities for people with disabilities based on life and career phase.

## Background

The rise in precarious work and erosion of standard employment opportunities are markers of the changing labor market, and represent critical social determinants of health in working populations [1, 2]. Labor market subgroups such as people living with disabilities may be at greater risk of working precariously. Also, research indicates that the relationship between precarious work can differ across age and according to an individual’s job tenure. Drawing from a large survey of Canadians employed for at least 15 h/week, the overarching objectives of our paper is to compare the likelihood of working precariously in a sample of adults living with and without a disability, and to examine how disability can modify the association between age and job tenure and precarious work. Our study provides insights into the socioeconomic conditions that may contribute to health inequities for people living with a disability [2].

Currently, there is no single definition or measure of precarious work that has been applied to people living with disabilities. Several conceptual frameworks exist in the literature that have primarily been applied to those not living with a disability [1, 3, 4]. A synthesis of existing conceptual frameworks indicate that precarious work is a heterogenous concept that can encompass uncertain (i.e., threat of job loss), unpredictable, unprotected and/or low paying employment where a person may have limited job control (i.e., jobs with high physical or mental demands and little control or influence over work) and/or regulatory protection (i.e., protection against unfair dismissal or unhealthy working conditions through organized labor groups such as unions) [1, 4, 5]. Those employed precariously also have less access to resources that sustain health (e.g., living wages, health insurance, paid sick leave, pension plan and social support) and are more likely to be exposed to workplace hazards when compared to those reporting more secure working conditions [6–10]. Within the literature, non-standard and contingent employment contracts including non-permanent (e.g., temporary and seasonal employment) and involuntary part-time work are often seen as a proxy for those who are more likely to work precariously [3, 4, 9]. Drawing from a synthesis of different conceptual models, we identify precarious workers as those employed in part-time work hours, in a non-permanent contract or where there is no union representation and where an employee reports low job control. The importance of studying precarious work is underscored by research that highlights its relationship to socioeconomic position and the likelihood of being exposed to risk factors for physical and mental morbidity [2, 11–13]. Understanding the factors associated with precarious work is important to developing policies and programs to address health inequities at the population level.

Research in Australia, Canada, the United States and elsewhere indicates that non-standard employment contracts are increasingly commonplace [3, 14, 15]. Contributing factors to rising workplace precarity include sociopolitical forces (e.g., globalization, recessionary periods, technological innovation) coupled with changes to the nature of work (e.g., gig work or jobs filled by temporary-help agencies) [5, 9]. Population-level studies in Canada, where the current research was conducted, indicate that between 1981 and 2018, the proportion of working-aged women and men in permanent full-time employment fell by 4 and 7%, respectively. During the same period, the proportion of those working in temporary or contract-type jobs increased from 46 to 53% [16]. Personal and organizational factors can also determine one’s likelihood of working precariously; women and those employed in highly physical and mentally demanding jobs are more likely to work precariously [17].

According to the World Health Organization’s (WHO) biopsychosocial model, a disability can be defined as any impairment in body function or structure that may interact with personal characteristics and/or socioenvironmental conditions to restrict participation in roles like employment [18]. Studies consistently indicate that people living with disabilities face challenges finding and sustaining paid work and advancing within the labor market, leaving them potentially more susceptible to precarious work [19–21]. People with disabilities are more likely to work part-time hours (< 30 h/week), be in short-term contracts, and earn less income [20–22]. Population-level data indicate that over one-third (36%) of Canadians with disabilities attribute their part-time work hours to their disability and 23% report being unable to find full-time work opportunities due to their health [22]. Additionally, studies indicate that people with disabilities are more likely to be employed in work environments where they are exposed to poor psychosocial conditions that include less control over their ability to sustain paid work and manage the physical and mental job demands [23, 24]. Exposure to low-quality employment for people with disabilities has the potential to contribute to labor market exclusion and worse health outcomes [24]. Few studies have drawn on a multidimensional framework of precarious work to determine whether people living with disabling health conditions are more or less likely to work precariously when compared to their peers without a disability.

This study draws on an industrial gerontological perspective. According to the perspective, different dimensions of temporality – including age and job tenure – can shape one’s experiences in the labor market and should be considered as separate constructs in studies of employment [25, 26]. To date, most research on precarious work has focused on the age-related differences. These studies indicate that young adults tend to start their careers in contingent jobs [27]. As they enter middle age, wokers are more likely to be employed in full-time permanent jobs [27]. Data from Canada’s General Social Survey indicates that young adults are more likely to work in lower quality jobs (e.g., insecure, unmanageable workloads and/or irregular schedules) when compared with middle- and older-aged adults who are more likely to work in secure permanent employment [27, 28]. Also, job tenure could be related to the likelihood of working precariously [29]. Canadian studies show that regardless of age, workers with shorter job tenures are more likely to report contingent and non-standard employment when compared to those with longer job tenures [30]. Other research in the field of occupational health and safety indicate that workers newer to an organization report less job security, greater exposure to physical and psychosocial workplace hazards and have a higher likelihood of work injury when compared with those reporting longer job tenures [29, 31]. Currently, there exists a limited body of research that has examined whether a disability modifies the relationships between precarious work, age and job tenure. As a result, we lack evidence needed to inform the development of tailored strategies that can be utilized to support the employment of people with disabilities at different life and career phases.

Our study objectives are to compare the likelihood of reporting precarious work in sample of employed adults living with or without a disability, and to examine the role of disability as a moderator in the relationships between precarious work, age and job tenure. We hypothesize that:

1. Employed people living with a disability will be significantly more likely to report precarious work than those not living with a disability.
2. Younger adults will be significantly more likely to report precarious work compared to middle- and older-aged adults. The relationship between being a young adult and precarious work will be modified by disability status. The likelihood of working precariously for young adults with a disability will be greater than for young adults not living with a disability.
3. Participants with shorter job tenures will be significantly more likely to report precarious work than those with longer job tenures. The relationship between shorter job tenure and precarious work will be modified by disability status. The likelihood of working precariously for those with a shorter job tenure who report a disability will be greater than those with a shorter job tenure not living with a disability.

## Methods

An online survey of Canadians living with and without a disability was conducted in August 2018. Eligible participants were 18 years of age and fluent in English and were employed for pay for at least 15 h/week. Given the challenges associated with recruiting large community-based samples of employed people with disabilities [32], potential survey participants were purposively recruited from an existing panel that is maintained by a research firm. A targeted recruitment approach was used to identify participants from the panel who were living with or without disabilities and across different age groups. The panel consisted of over one million Canadians and is nationally representative according to region and income [33]. Potential participants identified from the panel were contacted, provided study information and asked to complete a short screening questionnaire to determine eligibility. For those who chose to participate, informed consent was obtained, and the full online survey was administered. All study procedures were approved by the University of Toronto research ethics board (REB# 36184).

### Survey measures

The survey was developed by the research team. Items and measures were selected based on their feasibility and evidence of precision, validity and reliability in previous studies of people with and without disabilities. When no existing item or measure existed, new ones were developed.

### Outcome: precarious work

At the time of survey development, the research team were unable to identify any established measurement tools to examine precarious work that had been validated among people with disabilities. For the purpose of this study, we developed a specific measure of precarious work that draws directly from a synthesis of existing conceptual models and is relevant to people with and without disabilities [1, 3, 4]. Our measure included the following four items (see Appendix, Table S1):

#### Work hours

Using an open-ended question, participants were asked about the number of hours they worked per week. Respondents working < 30 h/week were categorized as working part-time and those working ≥ 30 h/week were categorized as working full-time [34].

#### Employment contract

Using one item developed for the survey, participants were asked if they were currently employed in a permanent position (i.e., no limit to duration) or a non-permanent position (i.e., limited duration in contract).

#### Job control

Participants were asked the following item “To what extent do you have control over your work schedule and how you do your work?”. Item response was on a five-point Likert scale (1 = not at all; 5 = a great deal). Those reporting ‘not at all’ or ‘a little’ job control were categorized as having low job control.

#### Union membership

One item, developed for this study, was used to assess whether participants belonged to a union and receive regulatory protection (1 = yes; 0 = no).

Using each item, participants were categorized as working precariously when they were employed in part-time hours *and/or* were working in a non-permanent contract *and/or* had no union representation *and* reported low job control.

### Predictor variables

#### Disability

We utilize an adapted version of the Disability Screening Questionnaire (DSQ), which was designed by Statistics Canada to identify people living with a disability within population health surveys [35]. The DSQ is based on the WHO biopsychosocial model of disability to identify individuals who face activity limitations related to five disability categories [35]. In our survey, five items were used to ask participants about the extent to which they face difficulties at work that have lasted or are expected to last for 6 months or more and are related to a physical, cognitive, mental/emotional, sensory, or other disability. Item response occurred on a four-point scale (0 = no; 1 = some; 2 = often; 3 = always). Participants who reported at least ‘some’ difficulty on at least one item were categorized having a disability [35]. The DSQ has been extensively psychometrically tested and has exhibited reliability and validity [35].

Drawing from an industrial gerontological framework, both age and job tenure are examined as separate predictor variables [25, 26].

#### Age

Based on their age, participants were divided into: young (18–35 years), middle-aged (36–50 years) and older adult (> 50 years) groups.

#### Job tenure

Number of years employed in current job.

### Covariates

Drawing from the WHO’s biopsychosocial model of disability and a large body of previous research, our analytical models adjusted for sociodemographic, health and work context factors [18, 36]. Specific covariates were selected when they were relevant to participants with and without disabilities.

#### Sociodemographic

Gender, educational attainment, marital status and personal income was collected.

#### Health factors

Participants were asked about their perceptions of their health using the widely utilized one-item self-rated health (1 = poor health; 5 = excellent health) [37]. Of note, self-rated health is a commonly used measure that can be applied to people with and without disability to capture global ratings of health and is seen as a powerful predictor of mortality and healthcare utilization [37, 38]. Additionally, pain and fatigue were also examined using visual analog scales (0 = no pain/fatigue; 10 = worst possible pain/fatigue) [39].

#### Work characteristics

Work characteristics were examined as covariates to account for different occupations and job roles of study participants.

Job sector (e.g., business/administration, health/science/teaching, sales/service, and trades/ transportation sectors) and organizational size were collected (e.g., small [1–50 people], medium [51–150 people] and large [> 150 people]). Two questions asked about the extent of physical and mental work demands (1 = not at all; 5 = a great deal). To assess productivity loss, one item from the Work Productivity and Activity Impairment instrument was utilized. Participants were asked the extent to which their health affected their job in the last month (0=“health had no effect on my work”; 10=“health completely prevented me from working”). The item is a valid and reliable tool to examine lost productivity attributed to disability [40].

### Analyses

Descriptive statistics (i.e., frequencies and means) were used to build a profile of the study sample and to examine variable distributions. Bivariate analyses (chi-square and t-tests) were conducted to examine how study variables differed between those with and without a disability.

Univariable logistic regression models were conducted for the total sample and for those with and without a disability to examine the association between predictor variables and study covariates and the likelihood of reporting precarious work. For multivariable modelling, covariates that were significantly associated with employment in the univariable model and did not exhibit multicollinearity with other covariates or the outcome variable were carried forward. To test study hypothesis one, a multivariable logistic regression model was conducted to examine the relationship between disability and precarious work when adjusting for study covariates.

To test study hypotheses two and three, a multigroup probit model using weighted least square mean and variance adjusted estimation (WLSMV) was conducted to examine the relationships between age and job tenure and precarious work, as well as to examine the modifying effect of disability. Multigroup modeling tests similarities and differences in coefficients of interest across a grouping variable (i.e., disability). To develop a multigroup model, a partially constrained model (i.e., parameter of interest differs between those with and without a disability) is compared to a fully constrained model (i.e., parameters are fixed across those with and without a disability). Through this approach, the modifying effect of disability can be determined. In our study, separate partially constrained models were conducted where all coefficients were constrained except for age (model a) and job tenure (model b). Also, separate models were also conducted for gender (model c) and physical job demands (model d) and mental job demands (model e) to account for their theoretical importance to precarious work. Each of the partially constrained models were then compared to a fully constrained model. Equality of coefficients across those with and without a disability were tested using the Satorra-Bentler scaled chi-square difference test for WLSMV implemented in Mplus [41].

Drawing from the findings from the multigroup probit model using WLSMV estimation, a final partially constrained multigroup probit model enabled the estimation of odds ratios. The multigroup probit model was stratified for those with and without disabilities. Also, age and job tenure, as well as gender and physical and mental job demands, were unconstrained in the final model; all other parameters were constrained to be equal. The multigroup model was also estimated using maximum likelihood parameter estimates with standard errors and a chi-square test statistic that was robust to non-normality. Analyses were conducted using SAS version 9.3 [42] and Mplus software [41].

## Results

Over eighteen hundred employed participants with (*n* = 901) and without a disability (n = 901) completed the survey. In line with recruitment strategies, almost equal proportions were young (18–35 years), middle-aged (36–50 years) and older-aged adults (> 50 years) (Table 1). Over half of the sample were women (56%) and married/living as if married (57%). Participants living with a disability less frequently reported graduating from post-secondary school when compared to those without a disability (48% vs 60%) and more often earned an income <$50,000 (41% vs 31%). Participants with disabilities reported greater pain and fatigue and lower self-rated health compared with those not living with a disability.

**Table 1:** Sample characteristics for the total sample, and compared by disability groups

	Total	No disability	Disability^**a**^	***P***
	Mean ***±*** SD/n (%)	Mean ***±*** SD/n (%)	Mean ***±*** SD/n(%)	
Number of participants	1802	901	901	
**Sociodemographic factors**				
Gender (women)	1011 (56.3)	494 (54.8)	517 (57.8)	0.20
Age (years)				0.99
Young adult (18–35)	597 (33.2)	301 (33.4)	296 (33.1)	
Middle-aged adult (36–50)	600 (33.4)	300 (33.3)	300 (33.5)	
Older adult (> 50)	599 (33.4)	300 (33.3)	299 (33.4)	
Marital Status				0.06
Married/living as married	1018 (56.8)	529 (58.9)	489 (54.7)	
Widowed/divorced/separated	217 (12.1)	94 (10.5)	123 (13.8)	
Never married	556 (31.0)	275 (30.6)	281 (31.5)	
Educational attainment				<.001
Primary-high school	334 (18.6)	142 (15.8)	192 (21.5)	
Some post-secondary	148 (27.5)	222 (24.7)	270 (30.2)	
Graduated post-secondary	965 (53.9)	534 (59.5)	431 (48.3)	
Income				<.001
< $50,000	607 (35.8)	260 (30.7)	347 (40.9)	
$50,000 – $89,999	624 (36.8)	321 (37.9)	303 (35.7)	
$90,000 ≤	465 (27.4)	266 (15.7)	199 (23.4)	
**Health factors**				
Pain (0–10)	2.6 (2.7)	1.4 (1.9)	3.9 (2.8)	<.001
Fatigue (0–10)	3.7 (2.9)	2.3 (2.4)	5.1 (2.8)	<.001
Self-rated health (1–5)	3.0 (1.0)	3.5 (0.9)	2.6 (0.9)	<.001
**Work characteristics**				
Job sector				0.21
Business/administration	419 (23.4)	220 (24.5)	199 (22.4)	
Health/science/teaching	609 (34.1)	314 (34.9)	295 (33.1)	
Sales/service	392 (21.9)	197 (21.9)	195 (21.9)	
Trades/transportation	369 (20.6)	168 (18.7)	201 (22.6)	
**Organization size (#of people)**				0.01
Small (1–50)	437 (25.5)	224 (25.8)	213 (25.2)	
Medium (51–150)	318 (18.5)	134 (15.4)	184 (21.7)	
Large (150<)	960 (56.0)	511 (58.8)	449 (53.1)	
Job tenure (# of years)	9.5 ***±*** 9.0	9.7 ***±*** 9.0	9.4 ***±*** 9.0	0.51
Perceived work physical demands (1-5)	2.9 ***±*** 1.4	2.7 ***±*** 1.4	3.1 ***±*** 1.4	<.001
Perceived work mental demands (1-5)	3.6 ***±*** 1.2	3.4 ***±*** 1.2	3.7 ***±*** 1.1	<.001
Productivity loss (0–10)	2.3 ***±*** 2.9	1.1 ***±*** 2.2	3.6 ***±*** 2.9	<.001
**Precarious working conditions**				
Precarious work (yes)	527 (29.7)	243 (27.3)	284 (32.1)	0.026
Contract type				
Permanent contract	1628 (90.9)	819 (91.2)	803 (90.4)	.57
Non-permanent contract	164 (9.1)	79 (8.8)	85 (9.6)	
Work hours				
Part-time (< 30 h/week)	205 (11.4)	92 (10.2)	112 (12.5)	.12
Full-time (≥ 30 h/week)	1596 (88.6)	808 (89.8)	783 (87.5)	
Union representation	552 (30.9)	250 (27.9)	302 (33.9)	<.001
Perceived Job control	2.8 ***±*** 1.2	2.9 ***±*** 1.2	2.7 ***±*** 1.2	<.001
Not at all	308 (17.2)	113 (14.9)	173 (19.5)	<.001
A little	453 (25.3)	213 (23.9)	237 (26.7)	
Somewhat	481 (26.9)	240 (26.9)	241 (27.1)	
Quite a bit	359 (20.1)	192 (21.5)	166 (18.7)	
A great deal	187 (10.6)	115 (12.9)	72 (8.1)	

Notes: *SD* Standard deviation, *n* number; ^a^Participants who reported activity limitations related to a physical, cognitive, mental/emotional, sensory impairment, or other disability

Similar proportions of participants with and without disabilities indicated working in a permanent job (90% vs. 91%) and full-time hours (88% vs. 90%). A greater proportion of participants with disabilities reported having union representation compared to those without a disability (34% vs. 28%) (*p* < .001). Participants with disabilities more frequently reported ‘none’ or ‘a little’ job control compared to respondents without a disability (46% vs. 39%) (*p* < .01). A mean job tenure of 9.5 years (SD = 9.0) was found across all survey respondents. Also, over one-third of all respondents worked in health/science/teaching job sectors and over half worked in large organizations (56%). Participants with disabilities reported significantly greater physical and mental demands and lost work productivity compared to participants not living with a disability (Table 1).

Univariable analyses was conducted to examine the relationship between each of the predictor variables and covariates and precarious work. At the univariable level, a significantly greater proportion of respondents with disabilities (32%) worked precariously than those without a disability (27%) (OR = 1.26; 95%CI 1.03,1.55) (Tables 1 and 2). Among participants not living with a disability, older adults had 62% lower odds of working precariously when compared young adults (OR = 0.62; 95%CI 0.43,0.90). For participants with (OR = 0.97; 95%CI 0.96,0.98) and without a disability (OR = 0.97; 95%CI 0.95,0.98) greater job tenure was significantly associated with a lower likelihood of working precariously.

**Table 2:** Univariable logistic regression examining the association between study variables and precarious work for the total sample and for those with and without a disability

	Total	No disability	Disability^**a;**^
	OR (95%CI)	OR (95%CI)	OR (95%CI)
**Independent variables**			
Disability status			
No disability	ref	–	–
Disability	1.26 (1.03, 1.55)		
Age (years)			
Young adult (18–35)	ref	ref	ref
Middle-aged adult (36–50)	0.95 (0.74, 1.21)	0.86 (0.61, 1.22)	1.07 (0.76, 1.52)
Older adult (> 50)	0.88 (0.69, 1.13)	0.62 (0.43, 0.90)	1.22 (0.87, 1.73)
Job tenure (years)	0.97 (0.96, 0.98)	0.97 (0.96, 0.99)	0.97 (0.95, 0.98)
**Sociodemographic**			
Gender (women)	1.23 (1.00, 1.52)	1.06 (0.79, 1.43)	1.42 (1.06, 1.90)
Marital Status			
Married/living as married	ref	ref	ref
Widowed/divorced/separated	1.63 (1.19, 2.22)	1.29 (0.79, 2.10)	1.86 (1.24, 2.80)
Never married	1.22 (0.97, 1.53)	1.33 (0.96, 1.84)	1.11 (0.81, 1.53)
Educational attainment			
Primary-high school	1.16 (0.89, 1.52)	1.12 (0.74, 1.69)	1.14 (0.80, 1.64)
Some post-secondary	0.99 (0.78, 1.26)	1.00 (0.70, 1.42)	0.95 (0.68, 1.32)
Graduated post-secondary	ref	ref	ref
Income			
< 50,000	ref	ref	ref
$50,000 – $89,999	0.44 (0.35, 0.57)	0.52 (0.36, 0.75)	0.39 (0.27, 0.54)
$90,000 ≤	0.40 (0.30, 0.52)	0.46 (0.31, 0.68)	0.36 (0.24, 0.53)
**Health factors**			
Pain (0–10)	1.06 (1.02, 1.10)	1.13 (1.05, 1.21)	1.02 (0.96, 1.07)
Fatigue (0–10)	1.06 (1.03, 1.10)	1.08 (1.01, 1.14)	1.04 (0.99, 1.09)
Self-rated health (1–5)	0.78 (0.70, 0.86)	0.68 (0.58, 0.81)	0.86 (0.74, 1.01)
**Work characteristics**			
Job sector			
Business/administration	0.62 (0.45, 0.86)	0.55 (0.34, 0.89)	0.70 (0.46, 1.08)
Health/science/teaching	0.77 (0.58, 1.02)	0.94 (0.62, 1.44)	0.64 (0.43, 0.94)
Sales/service	1.48 (1.10, 2.00)	1.44 (0.92, 2.25)	1.57 (1.05, 2.36)
Trades/transportation	ref	ref	ref
Organization size (# of people)			
Small (1–50)	ref	ref	ref
Medium (51–150)	0.84 (0.61, 1.14)	0.86 (0.54, 1.37)	0.75 (0.50, 1.15)
Large (150<)	0.71 (0.56, 0.91)	0.70 (0.49, 0.98)	0.74 (0.52, 1.04)
Perceived work physical demands (1-5)	0.96 (0.89, 1.03)	0.84 (0.75, 0.94)	1.05 (0.95, 1.16)
Perceived work mental demands (1-5)	0.90 (0.83, 0.98)	0.80 (0.70, 0.90)	0.98 (0.87, 1.11)
Productivity loss (0–10)	1.02 (0.99, 1.06)	1.00 (0.93, 1.07)	1.01 (0.96, 1.05)

Notes: *OR* Odds ratio, *CI* Confidence interval, *n* number, *ref.* reference category; ^a^Participants who reported activity limitations related to a physical, cognitive, mental/emotional, sensory, and/or other disability

Predictor variables along with covariates that were significantly associated with precarious work at the univariable level were carried forward in the multivariable logistic regression model for the full sample (Table 3). When adjusting for sociodemographic, health and work characteristic covariates, disability was no longer significantly associated with precarious work (OR = 1.01 95%CI, 0.76, 1.34). Of note, greater self-rated health was associated with a lower likelihood of reporting precarious work (OR = 0.77 95%CI, 0.67,0.88).

**Table 3:** Multivariable logistic regression model examining the association between study variables and precarious work for the total sample

	Full sample
	OR (95%CI)
**Independent variables**	
Disability status	
No disability	Ref
Disability	1.01 (0.76, 1.34)
Age (years)	
Young adult (18–35)	ref
Middle-aged adult (36–50)	1.14 (0.85, 1.53)
Older adult (> 50)	1.14 (0.82, 1.60)
Job tenure (years)	**0.97 (0.96, 0.99)**
**Sociodemographic**	
Gender (women)	1.17 (0.91, 1.48)
Marital Status	
Married/living as married	ref
Widowed/divorced/separated	**1.57 (1.10, 2.25)**
Never married	1.20 (0.93, 1.56)
Educational attainment	
Primary-high school	0.96 (0.68, 1.34)
Some post-secondary	0.87 (0.66, 1.14)
Graduated post-secondary	ref
**Health factors**	
Pain (0–10)	1.06 (0.99, 1.13)
Fatigue (0–10)	1.03 (0.97, 1.09)
Self-rated health (1–5)	**0.77 (0.67, 0.88)**
**Work characteristics**	
Job sector	
Business/administration	**0.58 (0.40, 0.84)**
Health/science/teaching	0.72 (0.52, 1.01)
Sales/service	1.22 (0.86, 1.71)
Trades/transportation	ref
Organization size (# of people)	
Small (1–50)	ref
Medium (51–150)	0.74 (0.52, 1.05)
Large (150<)	0.81 (0.61, 1.07)
Productivity loss (0–10)	0.96 (0.91, 1.01)
Perceived work physical demands (1-5)	**0.90 (0.82, 0.98)**
Perceived work mental demands (1-5)	0.90 (0.81, 0.99)

Notes: *OR* Odds ratio, *CI* Confidence interval, *ref.* reference category; ^a^Participants who reported activity limitations related to a physical, cognitive, mental/emotional, sensory and/or other disability

A summary of the multigroup modeling process is presented in Table 4. Results of chi-square difference tests of each model where all parameters but the primary predictor variable is unconstrained is compared to a model where parameters are fully constrained are presented. Partially constrained models for age (model a: X^2^ (2) = 7.8, *p* < .01) and job tenure (model b: X^2^ (1) = 5.6, *p* < .01), our primary predictor variables, were significantly different when compared to the fully constrained model. Our results indicate that disability was a significant effect modifier in the relationships between age and job tenure and precarious work.

**Table 4:** Summary of multigroup modelling process including tests for equality of coefficients and partially constrained multigroup probit model

	Test for equality for separate constrained models	Multigroup analyses (Partially constrained model)^**‡**^	
		No disability	Disability^†^
	X^**2**^ (df)	OR (95%CI)	OR (95%CI)
**Independent variables**			
Age (years)	7.8 (2)^model^ ^a^*		
Young adult (18–35)		ref	ref
Middle-aged adult (36–50)		0.99 (0.66, 1.50)	1.31 (0.86, 1.99)
Older adult (> 50)		0.67 (0.42, 1.06)	**1.88 (1.19, 2.98)**
Job tenure (years)	5.6 (1)^model^ ^b^*	0.99 (0.97, 1.01)	**0.95 (0.93, 0.98)**
**Sociodemographic**			
Gender (women)	2.7 (1)^model^ ^c^	0.96 (0.69, 1.34)	1.36 (0.96, 1.91)
Marital Status			
Married/living as married		ref	ref
Widowed/divorced/separated		**1.56 (1.08, 2.24)**	**1.56 (1.08, 2.24)**
Never married		1.21 (0.93, 1.57)	1.21 (0.93, 1.57)
Educational attainment			
Primary-high school		0.95 (0.68, 1.34)	0.95 (0.68, 1.34)
Some post-secondary		0.87 (0.65, 1.14)	0.87 (0.65, 1.14)
Graduated post-secondary		ref	ref
**Health factors**			
Pain (0–10)		1.06 (0.99, 1.13)	1.06 (0.99, 1.13)
Fatigue (0–10)		1.03 (0.97, 1.09)	1.03 (0.97, 1.09)
Self-rated health (1–5)		**0.77 (0.68, 0.88)**	**0.77 (0.68, 0.88)**
**Work characteristics**			
Job sector			
Business/administration		**0.57 (0.39, 0.82)**	**0.57 (0.39, 0.82)**
Health/science/teaching		**0.70 (0.50, 0.98)**	**0.70 (0.50, 0.98)**
Sales/service		1.19 (0.84, 1.69)	1.19 (0.84, 1.69)
Trades/transportation		ref	ref
Organization size (# of people)			
Small (1–50)		ref	ref
Medium (51–150)		0.75 (0.52, 1.06)	0.75 (0.52, 1.06)
Large (150<)		0.79 (0.59, 1.05)	0.79 (0.59, 1.05)
Productivity loss (0–10)		0.95 (0.90, 1.01)	0.95 (0.90, 1.01)
Perceived work physical demands (1-5)	6.2 (1)^model^ ^d^*	**0.82 (0.72, 0.93)**	0.97 (0.86, 1.10)
Perceived work mental demands (1-5)	3.7 (1)^model ^^e^	**0.81 (0.70, 0.93)**	1.00 (0.85, 1.16)

Notes: *OR* Odds ratio, *CI* Confidence interval, *ref.* Reference category; † = Participants who reported activity limitations related to a physical, cognitive, mental/emotional, sensory and/or other disability; df = degrees of freedom; **‡** = all paths were constrained as equal accept for age (model a), job tenure (model b) as well as gender (model c), physical work demands (model d) and mental work demands (model e); * = partially constrained model is significantly different when compared to fully constrained model

A final partially constrained multigroup probit model was conducted where the predictor variables (i.e., age and job tenure) and those of theoretical importance (e.g., gender and physical and mental job demands) were unconstrained and all remaining parameters were constrained is presented on Table 4. For participants with disabilities, being an older-aged adult was significantly associated with 88% greater odds of working precariously when compared to young adults (OR = 1.88, 95%CI 1.19,2.98). Although not statistically significant, among participants not living with a disability, being an older adult was related to a lower likelihood of working precariously when compared to young adult participants (OR = 0.67, 95%CI 0.42,1.06). Additionally, for participants with disabilities, every additional year of job tenure was significantly associated with 5% lower odds of precarious work (OR = 0.95 95%CI 0.93, 0.98). The relationship between job tenure and precarious work was not statistically significant for respondents not living with a disability (OR = 0.99 95%CI 0.97, 1.01). The final model exhibited optimal fit (X^2^ = 11.3(13), *p* > .50, CFI and TLI indices = 1;RMSE =0.000) [43].

## Discussion

Precarity has become an inherent feature of industrialized labor markets and represents a significant population health concern that can contribute to health inequity [6, 11, 13, 23]. Our study is among the first to draw on a large sample of employed adults to better understand the relationship between disability and precarious work at different ages and according to a participant's job tenure. Interestingly, the relationship between disability and precarious work was not statisically significant in our multivariable model. However, disability modified the age-precarious work and job tenure-precarious work relationships. Being older and having a shorter job tenure was associated with precarious work for participants living with a disability. Results bring attention to the importance of policies and programs for older and new workers with disabilities to prevent precarious work and mitigate its potential health consequences.

One third of employed adult participants in our survey indicated that their work was precarious. In contrast to our first hypothesis, disability was not significantly related to precarious employment in the full multivariable model. Our research could further highlight that precarity increasingly characterizes industrialized labor markets and has the potential to affect all population segments [1, 5]. When adjusting for health and work context factors, exposure to precarious working conditions may not significantly differ for workers with and without disabilities. At the same time, people with disabilities have complex health and support needs that could be worsened by their working situations [21, 24]. Accordingly, precarious work has the potential to disproportionately affect people living with disabilities when compared to those without a disability. To build on study findings, research is needed to compare the economic and health-related impact of precarious work on people with and without disabilities. It is important to highlight that in our multivariable model, greater self-rated health was associated with a lower likelihood of reporting precarious work. It may be that for those with and without a disability, health perceptions may play a pronounced role in determining the likelihood of working precariously [44]. It could also be that those working in more stable and secure forms of employment are more likely to report better health [1, 3, 9, 12]. Additional longitudinal studies are needed to elaborate on the factors that contribute to precarious work for those with and without a disability.

Drawing from an industrial gerontological framework, we highlight the importance of temporality in the likelihood of working precariously. In particular, the likelihood of being exposed to non-standard and contingent employment contracts can differ according to a person’s age and job tenure [26]. Also, utilizing multigroup modeling, we found that disability was a significant effect-modifier in the relationships between precarious work and age (hypothesis two) and job tenure (hypothesis three). Results indicated that older respondents living with a disability were significantly more likely to work precariously. Findings did not support our second hypothesis, which posited that younger workers with a disability would be more likely to work precariously when compared to older adults with a disability. Advances in treatment and self-management have meant that people living with disabilities are living longer and working later into their lives [45]. Past studies of older workers indicate that a disability may disrupt one’s ability to perform workplace acts and tasks, and to keep up with the schedule and pace of work [45, 46]. Also, research on people not living with a disability also highlight less occupational mobility (i.e., perceived ability to change work) among older workers when compared to younger workers [29]. Drawing from previous research, the greater likelihood of older adults working precariously when compared to their younger counterparts could be attributed to disruption in person-job fit that could accompany a disability and a lower likelihood of changing their working situation. It’s important to highlight that existing research has focused mostly on the broader sociopolitical and workplace conditions that may determine the likelihood of working precarious. Our study underscores the need to unpack how the relationship between personal and health factors can impact the likelihood of working precariously.

In support of hypothesis three, the job tenure-precarious work relationship was significant for those with a disability. Past studies have found that new workers, independent of age, are more likely to report less job security, confidence managing job demands, access to workplace social support and perceived job satisfaction [31]. In addition, previous research indicates that people with disabilities who are new to a job experience challenges navigating the physical and psychosocial work environment, independently managing their health at work and are less likely to access accommodations and to receive opportunities for career advancement [21]. Although we cannot infer causality, our findings suggest that for those with a shorter job tenure, having a disability plays an important role in increasing the likelihood of working precariously. To enhance study findings, detailed evidence on the specific employment conditions experienced by new workers with disabilities is required to better understand why they may be more likely to work precariously, and to inform the development of workplace employment interventions that provide pathways for new workers with disabilities to transition out of precarious work to more stable employment arrangement.

Our study is one of the first to examine precarious work in a large sample of employed Canadians with and without disabling health conditions. We utilize multigroup modeling to examine the extent to which disability moderates the relationships between age and job tenure and precarious work. Study limitations include our cross-sectional study design, which prevented us from determining causality or understanding the work-related changes that people with disabilities may experience over time. Longitudinal research would enhance study findings and enable a further understanding of the effect of disability on precarious work and enhance insights on the work and life trajectories of people with disabilities, as well as the potential longer-term health effects of precarious work in our sample. Our categorization of precarious work draws from a synthesis of conceptual models and existing research and includes work hours, contract type, union protection and perceptions of job control. It is important to acknowledge that precarious work could also be conceptualized on a continuum and can capture range of additional aspects of a person's employment conditions (e.g., employment benefits, income benefits, scheduling predictability) [5]. Also, previous research indicates that part-time working arrangements of people with disabilities are often involuntary [24]. However, in our study, we did not measure the voluntariness of part-time work. Building on these limitations, we suggest that future studies are required to expand on the conceptualization and measurement of precarious work in people with disabilities. Lastly, although we recruited a large sample of employed participants with and without a disability across different age groups, we utilized a purposive sampling approach. Accordingly, our study may be limited in its generalizability to all employed Canadians with disabilities.

Nonetheless, our study has implications for the health and quality of life of people living with disabling health conditions. Exposure to precarious work can exacerbate personal, social and economic factors that contribute to health inequities and can create unpredictability in sustaining employment and accessing resources (e.g., income and health insurance) to support health across the life course [6, 47]. Results bring greater attention to the importance of policies and programs that address the potential public health consequences of precarious work for new workers and older adults who are also living with disabilities.

## Conclusions

Representing a critical public health issue, labor market precarity has the potential to widen health inequities, especially among vulnerable segments of the workforce. Our study further highlights the potential disadvantage that new and older workers living with disabilities face in the labor market when compared to their non-disabled peers. There is a requirement for additional research to expand on the relationship between disability and precarious work and better understand its long-term impact on health and quality of life. Importantly, we provide evidence in support of strategies that can address precarious work for people with disabilities.

## Supplementary Information

**Additional file 1: Table S1.** Survey items used to assess precarious work in study participants with and without a disability.

## Data Availability

The datasets generated and analysed in the current study are not publicly available to protect the privacy of research participants as outlined in the study’s research ethics protocol. Please contact the corresponding if you have questions about the availability of the data and study materials.

## Abbreviation

CI: Confidence interval

## References

[1] Benach J, Muntaner C (2007). Precarious employment and health: developing a research agenda. J Epidemiol Community Health.

[2] Burgard SA, Lin KY (2013). Bad jobs, bad health? How work and working conditions contribute to health disparities. Am Behav Sci.

[3] Lewchuk W (2017). Precarious jobs: where are they, and how do they affect well-being?. Econ Labour Relations Rev.

[4] Tompa E, Scott-Marshall H, Dolinschi R, Trevithick S, Bhattacharyya S (2007). Precarious employment experiences and their health consequences: towards a theoretical framework. Work.

[5] Vosko LF (2006). Precarious employment: towards an improved understanding of labour market insecurity. Precarious employment: understanding labour market insecurity in Canada. Edn. Edited by L.F. V.

[6] Benach J, Benavides FG, Platt S, Diez-Roux A, Muntaner C (2000). The health-damaging potential of new types of flexible employment: a challenge for public health researchers. Am J Public Health.

[7] Joyce K, Pabayo R, Critchley JA, Bambra C (2010). Flexible working conditions and their effects on employee health and wellbeing. Cochrane Database Syst Rev.

[8] Virtanen M, Kivimäki M, Joensuu M, Virtanen P, Elovainio M, Vahtera J (2005). Temporary employment and health: a review. Int J Epidemiol.

[9] Benach J, Vives A, Amable M, Vanroelen C, Tarafa G, Muntaner C (2014). Precarious employment: understanding an emerging social determinant of health. Annu Rev Public Health.

[10] Mouw T, Kalleberg AL (2010). Occupations and the structure of wage inequality in the United States, 1980s to 2000s. Am Sociol Rev.

[11] Ahonen EQ, Fujishiro K, Cunningham T, Flynn M (2018). Work as an inclusive part of population health inequities research and prevention. Am J Public Health.

[12] Rugulies R, Aust B, Burr H, Bültmann U (2008). Job insecurity, chances on the labour market and decline in self-rated health in a representative sample of the Danish workforce. J Epidemiol Community Health.

[13] Marmot M, Friel S, Bell R, Houweling TA, Taylor S (2008). Commission on social determinants of health: closing the gap in a generation: health equity through action on the social determinants of health. Lancet.

[14] Burgess J, De Ruyter A (2000). Declining job quality in Australia: another hidden cost of unemployment.

[15] Quinlan M, Mayhew C, Bohle P (2001). The global expansion of precarious employment, work disorganization, and consequences for occupational health: a review of recent research. Int J Health Serv.

[16] Temporary employment in Canada [https://www150.statcan.gc.ca/n1/pub/11-627-m/11-627-m2019034-eng.htm].

[17] Cranford CJ, Vosko LF, Vosko LF (2006). Conceptualzing precarious employment: mapping wage work across social location and occupational context. Precarious employment: understanding labour market insecurity in Canada.

[18] World Health Organization (2001). International classification of functioning, disability and health: ICF.

[19] Tompa E, Scott H, Trevithick S, Bhattacharyya S (2006). Precarious employment and people with disabilities. Precarious employment: understanding labour market insecurity in Canada. Edn. Edited by L.F. V.

[20] Statistics Canada (2017). A profile of persons with disabilities among Canadians aged 15 years or older, 2012. In*.* Ottawa, ON.

[21] Bonaccio S, Connelly CE, Gellatly IR, Jetha A, Ginis KAM (2019). The participation of people with disabilities in the workplace across the employment cycle: employer concerns and research evidence. J Bus Psychol.

[22] Turcotte M (2014). Persons with disabilities and employment. In: statistics Canada.

[23] LaMontagne AD, Krnjacki L, Milner A, Butterworth P, Kavanagh A (2016). Psychosocial job quality in a national sample of working Australians: a comparison of persons working with versus without disability. SSM-Popul Health.

[24] Milner A, Shields M, King T, Aitken Z, LaMontagne A, Kavanagh AM (2019). Disabling working environments and mental health: a commentary. Disabil Health J.

[25] Peeters M, van Emmerik H, Kooij D, de Lange A, Jansen P, Dikkers J. Older workers’ motivation to continue to work: five meanings of age. J Manag Psychol. 2008.

[26] Sterns HL, Doverspike D (1989). Aging and the training and learning process.

[27] MacDonald R, Furlong A (2016). Precarious work: the growing precarite of youth. Routledge handbook of youth and young adulthood.

[28] Chen WH, Mehdi T (2019). Assessing job quality in Canada: a multidimensional approach. Analytical studies branch research paper series.

[29] Cheng GHL, Chan DKS (2008). Who suffers more from job insecurity? A meta-analytic review. Appl Psychol.

[30] Government of Ontario (2017). **Vulnerable workers in precarious jobs**. The changing workplaces review edn. Toronto, ON.

[31] Breslin FC, Smith P (2006). Trial by fire: a multivariate examination of the relation between job tenure and work injuries. Occup Environ Med.

[32] Lysaght R, Kranenburg R, Armstrong C, Krupa T (2016). Participant recruitment for studies on disability and work: challenges and solutions. J Occup Rehabil.

[33] 2017 Panel Bool [http://corporate.askingcanadians.com/services/panel/].

[34] Labour force survey [https://www.statcan.gc.ca/eng/survey/household/3701].

[35] Grondin C (2017). Canadian survey on disability, 2012: a new survey measure of disability: the disability screening questions (DSQ).

[36] World Health Organization and The World Bank (2011). World report on disability.

[37] Idler EL, Benyamini Y (1997). Self-rated health and mortality: a review of twenty-seven community studies. J Health Soc Behav.

[38] Miilunpalo S, Vuori I, Oja P, Pasanen M, Urponen H (1997). Self-rated health status as a health measure: the predictive value of self-reported health status on the use of physician services and on mortality in the working-age population. J Clin Epidemiol.

[39] McDowell I, McDowell I (2006). Visual analogue pain rating scales. Measuring health.

[40] Reilly MC, Zbrozek AS, Dukes EM (1993). The validity and reproducibility of a work productivity and activity impairment instrument. Pharmacoeconomics.

[41] Muthén LK, Muthén BO (2011). Mplus Use’s guide.

[42] SAS Institute Inc (2015). SAS 9.3. In*.*, version 9.3 edn.

[43] Hu L, Bentler PM (1999). Cutoff criteria for fit indexes in covariance structure analysis: conventional criteria versus new alternatives. Struct Equ Model Multidiscip J.

[44] Lund T, Borg V (1999). Work environment and self-rated health as predictors of remaining in work 5 years later among Danish employees 35–59 years of age. Exp Aging Res.

[45] Gignac MA, Smith PM, Ibrahim S, Kristman V, Beaton DE, Mustard CA (2019). Retirement expectations of older workers with arthritis and diabetes compared with those of workers with no chronic diseases. Can J Aging.

[46] Perkins EA, Moran JA (2010). Aging adults with intellectual disabilities. Jama.

[47] Milner A, Krnjacki L, Butterworth P, Kavanagh A, LaMontagne AD (2015). Does disability status modify the association between psychosocial job quality and mental health? A longitudinal fixed-effects analysis. Soc Sci Med.

