# Supplementary material for: The working disadvantaged: the role of age, job tenure and disability in precarious work
Source: BMC Public Health. 2020 Dec 10;20:1900. doi: 10.1186/s12889-020-09938-1 (PMC7727192; doi:10.1186/s12889-020-09938-1)
Supplement: Supplementary file 1 — Additional file 1: Table S1. Survey items used to assess precarious work in study participants with and without a disability. [file 12889_2020_9938_MOESM1_ESM.docx]

**Table S1.** Survey items used to assess precarious work in study participants with and without a disability.

| **Item** | **Question stem** | **Response options** |
| --- | --- | --- |
| 1. Work hours | How many hours on average do you work per week? | Open-ended |
|  |  |  |
| 1. Employment contract | Do you have a permanent position with your employer or are you on a contract? | ❑ Permanent position  ❑ Contract position  ❑ Other |
|  |  |  |
| 1. Job control | To what extent do you have control over your work schedule and how you do your work? | ❑ Not at all  ❑ A little  ❑ Somewhat  ❑ Quite a bit  ❑ A great deal |
|  |  |  |
| 1. Union memberships | Do you belong to a union or a professional/managerial society at your place of employment that acts as a bargaining unit? | ❑ No  ❑ Yes  ❑ Other |
